# Dental medical education in Croatia

**DOI:** 10.3325/cmj.2011.52.663

**Published:** 2011-12

**Authors:** Dragutin Komar

**Affiliations:** Dean of the University of Zagreb School of Dental Medicine

The University of Zagreb School of Dental Medicine is the only University School in Croatia that offers integrated undergraduate, graduate, specialist, and doctoral studies in Dental Medicine. It is one of the leading institutions in the country involved in teaching, scientific, and professional activities in dental medicine.

Education of doctors of dental medicine requires the application of the most recent knowledge, technologies, and materials. Therefore, we strive to innovate and search for the most efficient practical solutions and research initiatives in order to keep up with the standards of the European higher-education system.

Our priority is educating young professionals to become experts and scientists who will be able to face the professional challenges of the European higher-education system. Our institution needs to have a clear research profile characterized by research excellence acknowledged at the international level and evidence-based teaching program oriented to the future. By developing new ideas and technological solutions and by applying critical thinking, we would like to promote knowledge and creativity, which we believe are the foundations of the societal well-being.

Next year, 2012, the School marks its 50th anniversary and ever since its establishment one of our primary goals has been the systematic promotion of research in dental medicine. By taking part in various research projects, the Dental School strives to enhance the quality of research and research-based teaching. We wish to build stronger connections with the international community to foster scientific excellence and achieve visibility and recognition.

In order to improve research productivity and quality of research we continuously increase the number of research fellows, and doctoral and post-doctoral students. We take part in a growing number of research projects, especially international ones, and in parallel we increase the investment into infrastructure to ensure the organizational and financial framework required to implement these goals. Many young experts educated at our School successfully continued their education at many universities and institutes all over the world, and especially within the European system of higher education.

The inclusion of three research articles on dental medicine into this issue of the *Croatian Medical Journal* represents an important confirmation of the quality of our work.

The epidemiological study conducted by Walter Dukić et al (1) found a high prevalence of caries in children in Zagreb, confirming that long-term effectiveness of the system can only be ensured by placing an emphasis on preventive activities. This reminds us how important it is to distinguish the basic dental care, which every developed society should provide to its citizens, from the non-compulsory one, which is the responsibility of an individual. Prevention is the foundation of each nation’s health care system and it should be strengthened by adequate health policies in order to avoid future complications. It is precisely by encouraging prevention that the most developed European countries reduce the need for treatment procedures. The implementation of this concept requires young highly educated staff and the production of such professionals is the primary task of the School.

The article by Jurica Matijević et al (2) shows that we still cannot be completely satisfied with the quality of endodontic treatment provided in Croatia, because the incidence of apical periodontitis in treated teeth is too high. This points out that continuous, lifelong learning and a transfer of new knowledge and technologies is the basic precondition for quality professional work of dentists.

Romana Peršić et al (3) showed that Croatian dentists obtained similar results as their Austrian colleagues, which is a quite an encouraging finding. All of this confirms that we are on the right path in raising the educational, scientific, and professional standards. The inclusion of these three articles from the field of dental medicine into this issue of the *Croatian Medical Journal* is a further acknowledgement of the excellent work done by our scientists, as well as the schools in which they have acquired their skills, knowledge, and experience.

## References

[1] Dukic W, Delija B, Lulic Dukic O (2011). Caries prevalence among school children from Zagreb, Croatia.. Croat Med J.

[2] Matijevic J, Cizmekovic Dadic T, Prpic Mehicic G, Anic I, Slaj M, Jukic Krmek S (2011). Prevalence of apical periodontitis and quality of root canal fillings in the population of Zagreb, Croatia.. Croat Med J.

[3] Persic R, Kqiku L, Brumini G, Husetic M, Pezelj-Ribaric S, Brekalo Prso I (2011). Difference in the periapical status of endodontically treated teeth between the samples of Croatian and Austrian adult patients.. Croat Med J.

